# Polypharmacy and pharmacogenomics in high-acuity behavioral health care for autism spectrum disorder: a retrospective study

**DOI:** 10.1186/s13034-025-00915-3

**Published:** 2025-05-21

**Authors:** Sheldon R. Garrison, Sophie A. Schweinert, Matthew W. Boyer, Maharaj Singh, Sreya Vadapalli, Jeffery M. Engelmann, Rachel A. Schwartz, Madeline M. Hartig

**Affiliations:** 1 Rogers Behavioral Health, Research Center, 34700 Valley Road, Oconomowoc, WI 53066 USA; 2 Rogers Behavioral Health, Philadelphia, PA USA

**Keywords:** Autism, CYP2D6, ASD, Depression, Anxiety, Pharmacogenomics, PGx, GeneSight, Polypharmacy, Antipsychotics, Antidepressants

## Abstract

**Background:**

This study evaluated pharmacogenomic (PGx) testing in children and adolescents with autism spectrum disorder (ASD). ASD frequently presents with co-occurring depression and anxiety. This complex phenotype often results in psychotropic medication polypharmacy. Incorporating PGx testing into the medical work-up may reduce polypharmacy and improve quality of life with symptom reduction.

**Methods:**

A retrospective electronic health record (EHR) review between January 2017 and May 2023. Individuals either received PGx testing or treatment as usual (TAU). The co-primary outcomes were instance of polypharmacy and the Pediatric Quality of Life Enjoyment and Satisfaction Questionnaire (PQ-LES-Q). Secondary outcomes included length of stay, average number of psychotropic medications, readmissions and assessments measuring severity of symptoms or behavioral impact. When at least one daily psychotropic medication was prescribed and reported to have an increased probability of gene–drug interactions, the individual was classified as “incongruent” (PGx-I). Individuals were categorized as “congruent” (PGx-C) if all prescribed psychotropic medications were without potential gene–drug interactions. Polypharmacy was evaluated and compared within the PGx-C and PGx-I subgroups.

**Results:**

A total of 99 individuals with ASD were analyzed. At the time of admission, 93% of individuals were prescribed at least one psychotropic medication and over half of these individuals were prescribed medications with potential gene–drug interactions. Following PGx testing, there was an overall reduction in prescribed medications with potential gene–drug interactions. No differences were observed between the PGx and TAU groups in polypharmacy, quality of life, or symptom assessments of depression, anxiety, obsessive–compulsive disorder and body-focused repetitive behaviors. Subanalysis comparing congruent (“use as directed”) or incongruent (“use with caution”), as well as exploratory analysis of only CYP2D6 and CYP2C19 gene–drug interactions, were observed to have a similar profile between treatment groups for all primary and secondary outcomes, except for the average number of psychotropic medications prescribed.

**Conclusions:**

Incorporating PGx testing into the medical workup did not improve outcomes, with all treatment groups achieving similar levels of polypharmacy and quality of life. Analysis of secondary outcomes revealed some differences in medication prescribing when stratifying by congruency; however, no differences were observed between treatment groups for all other secondary outcomes.

**Supplementary Information:**

The online version contains supplementary material available at 10.1186/s13034-025-00915-3.

## Introduction

Autism spectrum disorder (ASD) is a neurological and developmental disorder affecting an estimated 2.9% of the population [43]. Symptoms typically manifest before age three and include deficits in communication and social interaction, repetitive behaviors, fixated interests and inflexible routines [2]. Depression, anxiety disorders, obsessive–compulsive disorder (OCD), attention-deficit/hyperactivity disorder (ADHD), and tics also frequently co-occur with ASD [23, 27]. Medication management for individuals with ASD often includes psychotropic medication polypharmacy, even in young children, underscoring the need to optimize medication selection to increase efficacy and minimize side effect burden.

Medication management for individuals with ASD can be complicated, with only aripiprazole and risperidone approved to treat irritability associated with ASD in children and adolescents. Moreover, no pharmacologic treatments that target the underlying etiology of ASD are approved. Without medications approved to target the ASD-specific etiology, multiple psychotropic medications are frequently prescribed to manage both ASD-related symptoms and those associated with co-occurring psychiatric conditions, such as antidepressants. Additionally, children with ASD often experience more adverse effects and show limited response to psychotropic medications compared to children not diagnosed with ASD [6, 8, 32]. This is further complicated by underlying clinically significant genetic variations affecting an individual’s neurophysiology and the heterogeneous symptomatic presentation among individuals with ASD, which often requires psychotropic polypharmacy with medications across different drug classes [41], including antidepressants, antipsychotics, mood stabilizers and stimulants.

One approach to streamline medication trialing and optimize dosing has been to incorporate pharmacogenomic (PGx) testing [9]. More specifically, PGx tests analyze genetics to predict pharmacokinetic (PK) and pharmacodynamic (PD) response to medications to address the limited response rates to first-line medications, which are reported to range from 42 to 53% in depression alone [11, 26]. Thus, PGx-guided drug selection and dosing may help to reduce medication side effects [18] and improve medication compliance [40], and thereby reduce the need for medication changes. PGx testing may also lead to reduced emergency room visits, readmissions and cost [13, 16, 19] and may be particularly relevant to persons receiving treatment at higher levels of care, such as in residential, partial hospitalization and intensive outpatient behavioral healthcare treatment programs. However, PGx remains controversial, as some studies report mixed or no clinical benefit [20], Evidence supporting PGx utility in child and adolescent populations is also limited. As a result the American Academy of Child and Adolescent Psychiatry (AACAP) recommends clinicians avoid using PGx testing to select psychotropic medications in children and adolescents [35].

Emerging data has demonstrated that when used for individuals with ASD, PGx testing can provide clinically meaningful information to guide medication management and improve clinical outcomes [46]. This includes global improvement in clinical symptoms when PGx is incorporated into medication management decisions for those with ASD who failed to respond to at least two psychotropic medications [4]. Additional studies for PGx-guided psychotropic medication management have followed guidelines by Clinical Pharmacogenetics Implementation Consortium (CPIC), the American Psychiatric Association Council of Research (APA-COR) or the Association for Molecular Pathology Clinical Practice Committee's Pharmacogenomics (PGx) Working Group and others [7, 21, 34, 48]. These focus on specific PK-related enzymes that are effective in ameliorating some ASD symptoms, including CYP2D6, which is reported to play a role in the metabolism of aripiprazole and risperidone [7, 34]. However, there may be limitations in interpreting how specific enzyme variants will affect safety and efficacy, particularly for medications with multiple active moieties, such as risperidone. For example, CYP2D6 metabolizes risperidone to 9-hydroxyrisperidone, which is an active metabolite but with decreased brain penetration. For individuals who are poor metabolizers at CYP2D6, the ratio of plasma risperidone to 9-hydroxyrisperidone may be increased, which could result in risperidone-related adverse effects without meaningfully affecting efficacy. Conversely, ultrarapid metabolizers at CYP2D6 may result in decreased plasma levels of risperidone, and as a result, may have decreased efficacy [25, 28, 44].

Genetic variants of multiple PK-related enzymes may confer ultra-rapid, intermediate or poor metabolism of select psychotropic medications. Taking these medications may result in poor therapeutic response to altered plasma concentrations and delayed drug clearance [7]. Although these genetic variants occur frequently in individuals with ASD [5, 17], there are currently no ASD-specific PGx guidelines for commercially available PGx products [5]. As a result, PGx utilization continues to be limited for ASD and continued investigation is warranted. Therefore, a retrospective study analyzing 99 persons diagnosed with ASD was conducted to determine whether PGx-guided medication management would improve medication congruence with potential gene–drug interactions, reduce polypharmacy and decrease symptom severity.

## Methods

### Participants and procedure

A retrospective electronic health record (EHR) review was conducted for children and adolescents with ASD treated acutely in an anxiety and depression partial hospitalization (PHP) and intensive outpatient (IOP) programs within a national behavioral health care system was approved by an institutional review board (IRB). Admission to this program required an ASD diagnosis. At admission, all available clinical information, assessments and evaluation with a child and adolescent psychiatrist and clinical psychologist were utilized to develop an individualized treatment plan, including medication and therapy planning. While standardized ASD-specific severity measures were not administered due to the type of program, the clinical information typically included diagnostic evaluations, family and school reports, neuropsychological assessments and external electronic medical records. Participants completed daily treatment within the PHP program (6–7 h of treatment daily) or the IOP program (3 h daily). Treatment included cognitive-behavioral therapy (CBT) with ASD-specific modifications, such as an emphasis on intrusive and inflexible thoughts, and social communication. Individuals in the program were able to participate in group-based CBT and other behavioral therapy practices. Overall, the programs were individualized and tailored to treating the anxiety and depression symptoms that were secondary to ASD. The goal of these specialized programs was to help improve overall quality of life, as opposed to treating the symptoms of ASD.

PGx testing was ordered on a case-by-case basis at the prescriber’s discretion, although 13% (6/46) of the PGx reports were provided by individuals or their parent/guardian at the time of admission. All eligible individuals with PGx testing reported between January 2017 and May 2023 were included in the study. Individuals included in the current study received daily behavioral therapy as part of their treatment program.

### Classification of medication congruence with PGx testing

Any psychotropic medication-related PGx test was eligible for inclusion in this study; however, all extracted reports for this study were the GeneSight^®^ Psychotropic test (Myriad Genetics, Inc., Salt Lake City, UT). Data were extracted from individual PGx reports and analyzed at the individual level. Determinations of gene–drug interactions were based on both pharmacokinetic (PK) and pharmacodynamic (PD) genes. The PGx treatment group was classified as “incongruent” (PGx-I) when individuals were prescribed at least one daily psychotropic medication that was reported to have a “moderate” or “significant” potential gene–drug interaction, or Individuals were classified as “congruent” (PGx-C) when prescribed medications that were all reported with use as directed, or “normal”, with no suspected gene–drug interactions. Individuals were also classified as congruent when medications in the moderate and significant categories were discontinued or titrated down before discontinuing following the PGx report.

Exploratory analysis was manually conducted to include only the CYP2D6 and CYP2C19 enzymes, which are two clinically actionable genes specifically referenced by CPIC, the Food and Drug Administration (FDA) and other consortia [7, 31]. CPIC has recognized CYP2D6 and CYP2C19 as specific genes that may be evaluated as part of the psychotropic medication selection decision, particularly around certain selective serotonin reuptake inhibitors (SSRI), serotonin-norepinephrine reuptake inhibitors (SNRI) and atypical antipsychotics due to the potential of increased side effect risk and reduced efficacy [7]. Analysis for the current study was kept broad to include any psychotropic medication metabolized by CYP2D6 or CYP2C19. Accordingly, if the PGx report indicated either gene as having an ultrarapid, poor or intermediate metabolizer status for at least one prescribed psychotropic medication it was classified as incongruent (CYP2D6/CYP2C19–I). If the report indicated ‘normal’ or ‘not reported’ for these genes, individuals were classified as congruent (CYP2D6/CYP2C19-C). The frequency of metabolizer status for these enzymes is included in Table S1 with outcomes included in Table S2.

### Outcomes

Two co-primary outcomes were evaluated for this study. The first co-primary outcome was the average number of medications prescribed following the PGx report, with a focus on polypharmacy. Due to the lack of a consensus definition for polypharmacy [45], two criteria were used to evaluate the impact of PGx testing on psychotropic polypharmacy. Polypharmacy was defined as: (1) three or more concurrent psychotropic medications or (2) two or more medications prescribed from the same class (e.g., serotonin reuptake inhibitor). Non-psychotropic medications were not reported. The second co-primary outcome was the Pediatric Quality of Life Enjoyment and Satisfaction Questionnaire (PQ-LES-Q). For the PQ-LES-Q, respondents were scored as a percentage of the total possible score and categorized as a low-quality of life (< 65%), average quality of life 65–83%, and high quality of life (84% and above) [3, 14]. The secondary outcomes included rate of length of stay (LOS), average number of psychotropic medications and symptomatic assessments. The symptomatic assessments included:

1. Patient-Reported Outcomes Measurement Information System Pediatric Depressive scale (PROMIS-D) was used to measure depression severity. Individuals rate the degree to which they experienced a given symptom (e.g., “I felt sad”) over the past week from 0 (*never*) to 4 (*almost always*), such that total scores range from 0 to 32 with higher scores reflecting greater depression [10].
2. Liebowitz Social Anxiety Scale for Children and Adolescents (LSAS-CA) is a 24-item self-report that assesses social anxiety severity [29].
3. Repetitive Body-Focused Behavior Scale (RBBS). The RBBS is broken down to assess the presence, severity, and associated consequences of skin picking, nail biting, and hair pulling [47].

The exploratory outcomes included the following and were reported in Table S3.

1. Penn State Worry Questionnaire for Children (PSWQ-C) is a 14-item self-report that assesses generalized worry [33].
2. Childhood Anxiety Sensitivity Index (CASI) is a self-report assessment of anxiety sensitivity on a scale ranging from 18 to 54 [1].
3. Children’s Yale-Brown Obsessive–Compulsive Scale (CY-BOCS) is an assessment designed to rate the severity of obsessions and compulsions using a scale ranging from 0 to 40. OCD symptom severity was scored as subclinical (0–7), mild (8–15), moderate (16–23), severe (24–31) and extreme (32–40) [12, 37].

### Matching

In this study, all individuals in the anxiety and mood disorders program for children and adolescents with ASD with PGx testing were included. Records were excluded if individuals discharged prior to the return of the PGx results. To get a randomized control group, treatment as usual (TAU), others in the same program were randomly selected using the MatchIt package within R, version 4.3.3 [22, 42]. Matching criteria included medical service line, ASD diagnosis and level of care, which was partial hospitalization or intensive outpatient so that a similar profile would be included within the TAU group.

### Statistical analysis

Categorical variables were described as count and percentages. Continuous variables were summarized as mean and standard deviation for normally distributed data, and as median and interquartile range for non-normally distributed data. The sample size (n) represented all individuals in the EHR meeting eligibility criteria. Group comparisons were performed using student’s t-test for independent samples for normally distributed continuous variables. Two-way repeated measures analysis of variance (ANOVA) were used for group (between-subjects factor) and time (admission to discharge; repeated measures factor), followed by Bonferroni post-hoc tests and Bonferroni correction for multiple comparisons. Either the Chi-Square or Fisher’s Exact test was used for categorical data comparisons, as appropriate. For all statistical tests, an alpha level of 0.05 was used to determine significance. Data were reported as means ± standard error of the mean (SEM) for continuous variables. All analyses were conducted using R software (version 4.3.3).

## Results

### Demographics and characteristics

There were no differences in demographic and clinical characteristics between the TAU and PGx groups (Table 1). The average length of stay (LOS) was similar between the TAU (37.1 ± 3.3 days) and PGx (36.7 ± 1.8 days) groups (P = 0.911). There was insufficient racial and ethnic diversity to report, with only individuals self-identifying as White and Non-Hispanic having ≥ 10 individuals per group. Quality of life at admission was low for both TAU (44.7 ± 1.7) and PGx (46.4 ± 1.8) groups (P = 0.664; Table 1), as assessed using the PQ-LES-Q. Depression scores at admission, measured using PROMIS-D, indicated moderate levels of depressive symptoms in both the TAU (15.3 ± 1.6) and PGx (14.5 ± 1.3) groups (P = 0.671; Table 1). LSAS-CA scores at admission reflected moderate levels of social anxiety for both the TAU (58.3 ± 5.5) and PGx (58.3 ± 5.2) groups (P ≥ 0.999; Table 1). The presence and severity of repetitive body-focused behavioral symptoms, measured using the RBBS, were similar between the TAU and PGx groups for skin-picking (P = 0.609), hair pulling (P = 0.382) and nail biting (P = 0.222) (Table 1).

**Table 1:** Demographic characteristics

	TAU	PGx	P value
n	53	46	
Age (y) (mean (SEM) [range])	14.2 (0.3) [10–20]	14.3 (0.4) [9–18]	0.819
Sex (%)			0.885
Female	39.6 (21)	41.3 (19)	
Male	60.4 (32)	58.7 (27)	
Level of care (%)			
Partial hospitalization	96.2 (51)	89.1 (41)	
Intensive outpatient	3.8 (2)	10.9 (5)	
Overall comorbidities (n)			
Comorbidities % (n)			
Anxiety disorders	96.2 (51)	93.5 (43)	
Attention-deficit/hyperactivity disorder	54.7 (29)	56.5 (26)	
Eating disorders	7.5 (4)	2.2 (1)	
Mood disorders	84.9 (45)	69.6 (32)	
Obsessive–compulsive and related disorders	41.5 (22)	63.0 (29)	
Post-traumatic stress disorder	9.4 (5)	6.6 (3)	
Tourette's disorder, Tic disorder	9.4 (5)	15.2 (7)	

Treatment response, comparison between groups at admission and discharge						
Variable [mean (SEM), n]	Admission			Discharge		
	TAU	PGx	P value	TAU	PGx	P value
PQ-LES-Q	44.4 (1.7), 42	45.4 (1.8), 39	0.664	52.4 (1.5), 22	51.8 (1.7), 26	0.805
PROMIS-D	15.3 (1.6), 32	14.5 (1.3), 38	0.671	8.1 (1.3), 27	10.5 (1.5), 24	0.223
LSAS-CA	58.3 (5.5), 32	58.3 (5.2), 38	0.999	34.5 (6.6), 19	43.5 (6.6), 25	0.339
RBBS						
Hairpulling	4.6 (1.3), 29	6.4 (1.4), 37	0.382	1.1 (0.6), 27	3.8 (1.5), 24	0.110
Skin picking	12.2 (2.1), 29	10.8 (1.8), 37	0.609	6.5 (1.6), 27	7.5 (1.9), 24	0.697
Nail biting	9.1 (1.6), 29	6.6 (1.3), 37	0.222	4.1 (1.1), 27	3.8 (1.2), 24	0.844
Length of stay (days) (SEM) [range]				37.1 (3.3) [1–130]	36.7 (1.8) [10–66]	0.911

Exploratory outcomes included the PSWQ, CASI and CY-BOCS assessments. Both the TAU and PGx groups had clinically elevated levels of worry at admission as measured with the PSWQ (P = 0.424; Table S3). Anxiety sensitivity, measured using CASI, revealed similarly elevated scores at admission in both TAU and PGx groups (P = 0.420; Table S3). Obsessive–compulsive disorder (OCD) symptoms, measured with the CY-BOCS, indicated that the symptoms at admission for both TAU and PGx groups were severe (P = 0.113; Table S3).

### Medication changes following PGx testing

PGx-guided psychotropic medication selection was anticipated to result in decreased polypharmacy and improved quality of life. Medications were frequently changed, or dosages adjusted, following the PGx report, particularly when the admitting medication had a potential gene–drug interaction. At the time of admission to either PHP or IOP levels of care, 96% (44/46) of individuals in the PGx group were already prescribed at least one psychotropic. Of these medications, 57% (26/46) of individuals were prescribed at least one incongruent medication. Following PGx testing, over half of those initially prescribed an incongruent medication had switched to a congruent medication by the time of discharge.

**Fig. 1:**
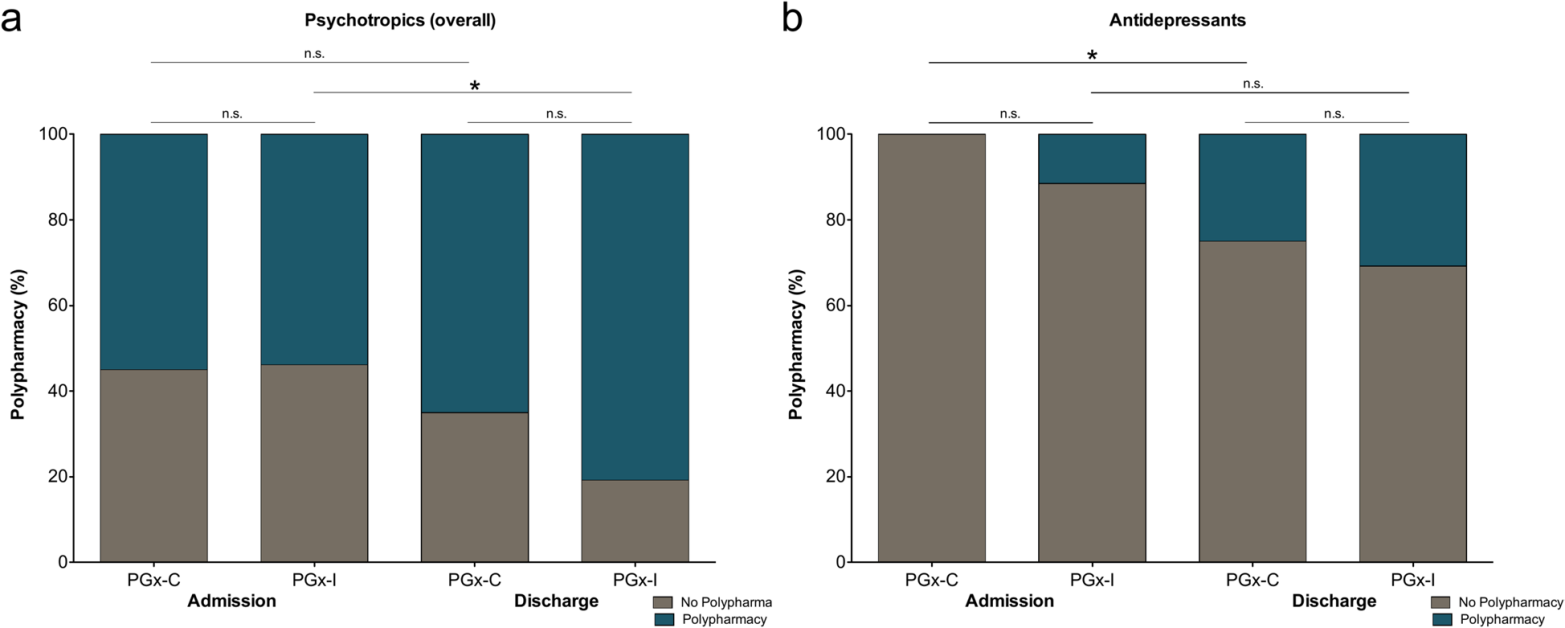
Polypharmacy rate between the PGx-C and PGx-I cohorts. **a** When evaluating all psychotropic medications at admission, the polypharmacy rate was similar between PGx-C and PGx-I. The PGx-C polypharmacy rate was 55.0% (11/20) and 53.8% for PGx-I (14/26) (P = 0.938). At discharge, the polypharmacy rate was also similar between PGx-C and PGx-I. The PGx-C polypharmacy rate was 65.0% (13/20) and 80.8% for PGx-I (21/26) (P = 0.227). The polypharmacy rate did not differ between admission and discharge for PGx-C (P = 0.519), but it did increase for PGx-I (P = 0.039). **b** When evaluating antidepressants at admission, the polypharmacy rate was similar between PGx-C and PGx-I. The PGx-C polypharmacy rate was 0.0% (0/20) and 11.5% for PGx-I (3/26) (P = 0.245). At discharge, the polypharmacy rate was also similar between PGx-C and PGx-I. The PGx-C polypharmacy rate was 25.0% (5/20) and 30.8% for PGx-I (8/26) (P = 0.667). The polypharmacy rate increased between admission and discharge for PGx-C (P = 0.047), but did not differ for PGx-I, with only a trend (P = 0.090). *P < 0.05; n.s. = not significant. Data reported as mean ± SEM

Multiple medications are often prescribed to treat the ASD-associated symptoms and symptoms of co-occurring psychiatric conditions. Polypharmacy was common in this cohort, with 53% of individuals prescribed three or more psychotropic medications at admission and 74% at discharge. Polypharmacy was then evaluated by analyzing individuals prescribed psychotropic medications congruent with their genetic profile (PGx-C) compared to individuals prescribed medications with probable gene–drug interactions (PGx-I). At admission, 55% of individuals met polypharmacy criteria in the PGx-C cohort compared to 54% in the PGx-I cohort (Fig. 1a; P = 0.938). Polypharmacy percentages increased to comparable levels for both groups by discharge, with 65% in the PGx-C cohort and 81% in the PGx-I cohort (Fig. 1a; P = 0.227). Differences in polypharmacy continued when stratifying by medication class. When evaluating antidepressants, polypharmacy rate was similar between PGx-C and PGx-I at admission Fig. 1b; P = 0.245); however, these cohorts separated by discharge with polypharmacy rate markedly higher in the PGx-I cohort compared to PGx-C (Fig. 1b; P = 0.667). Polypharmacy rates for the overall psychotropic medications and antidepressants for all subgroups were also calculated and reported in Table 2.

**Table 2:** Treatment response, comparison between PGx-Congruent and PGx-Incongruent at admission and discharge

Variable [mean (SEM), n]	Admission			Discharge		
	PGx-C	PGx-I	P value	PGx-C	PGx-I	P value
Age	14.7 (0.6)	14.1 (0.5)				
Polypharmacy % (n)						
All psychotropic medications	55.0	53.8	0.938	65.0	80.8	0.227
Antidepressants	0.0	11.5	0.254	25.0	30.8	0.667
PQ-LES-Q	41.4 (2.4), 16	48.2 (2.4), 23	0.059	49.1 (2.3), 11	53.7 (2.4), 15	0.188
PROMIS-D	17.3 (2.1), 15	12.6 (1.6), 23	0.079	12.5 (2.5), 10	9.1 (1.9), 14	0.295
LSAS-CA	64.9 (7.3), 15	54.0 (7.1), 23	0.309	48.2 (10.2), 11	39.9 (6.53), 14	0.483
RBBS						
Hairpulling	9.4 (2.8), 14	5.1 (1.5), 23	0.188	6.2 (3.2), 10	2.1 (1.2), 14	0.197
Skin picking	14.2 (3.0), 14	8.6 (2.2), 23	0.143	10.0 (3.5), 10	5.7 (2.1), 14	0.282
Nail biting	7.8, (2.5), 14	5.9 (1.4), 23	0.485	4.0 (2.6), 10	3.6 (1.1), 14	0.867

**Fig. 2:**
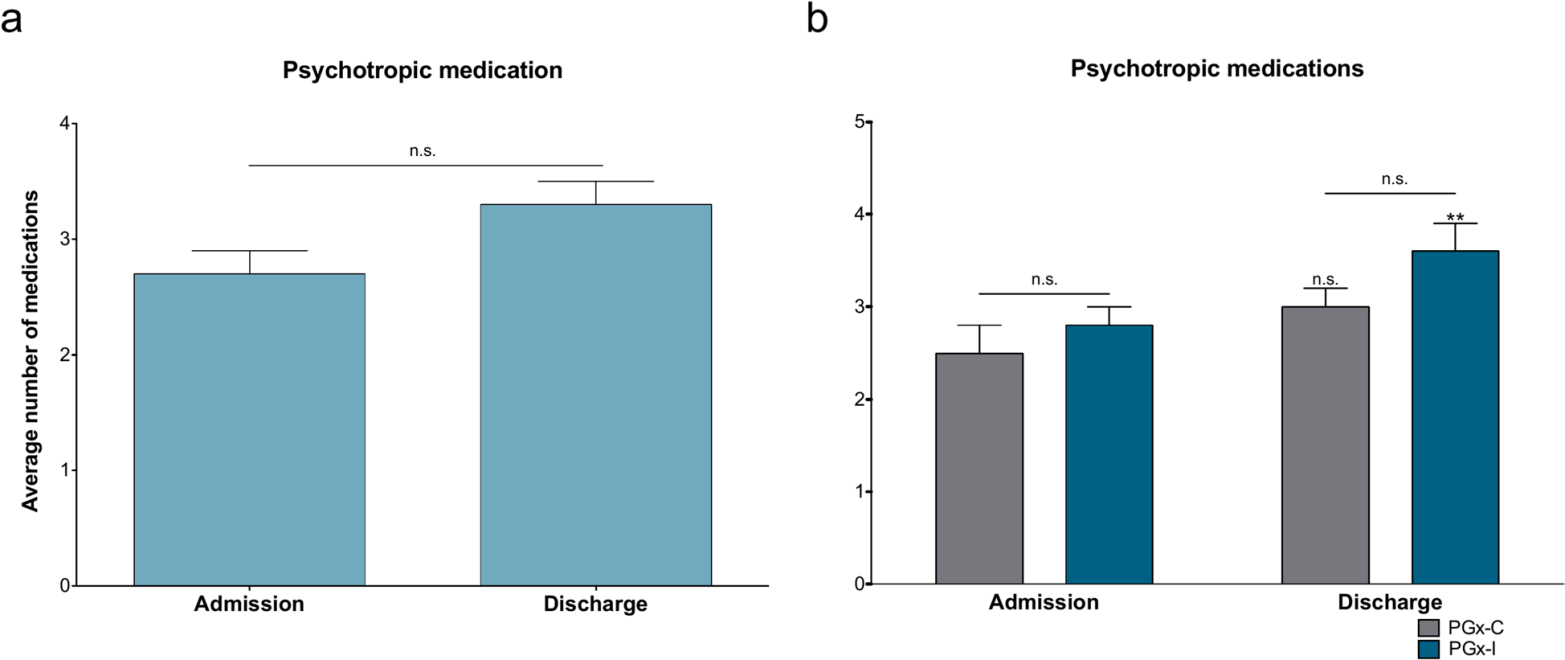
The average number of medications was similar between treatment groups. **a** Within the PGx group, the average number of medications increased between admission (2.7 ± 0.2) and discharge (3.2 ± 0.2) (P = 0.010). **b** At admission, the average number of medications did not differ between PGx-C (2.5 ± 0.3) and PGx-I (2.8 ± 0.2) (P = 0.450). At discharge the average number of medications did not differ between PGx-C (3.0 ± 0.2) and PGx-I (3.6 ± 0.3), although there was a trend (P = 0.115). The average number of medications did not differ between admission and discharge for PGx-C (P = 0.180), but they did increase for PGx-I (P = 0.027). n.s. = not significant. Data reported as mean ± SEM

While polypharmacy is an important metric for assessing psychotropic medication burden, it is limited by its binary nature and a more granular approach to understand differences in medication prescribing. This approach revealed that the average number of medications was 2.7 ± 0.2 at admission increased to 3.2 ± 0.2 at discharge (P = 0.011; Fig. 2a). At discharge the PGx-I cohort averaged 3.6 ± 0.3 medications, compared to 3.0 ± 0.2 for those prescribed congruent medications (PGx-C) (P = 0.115; Fig. 2b), which may be clinically meaningful but did not reach statistical difference. The PGx-I subgroup appeared to drive the increase in the average number of prescribed psychotropic medications from admission to discharge (P = 0.027), while the PGx-C subgroup remained relatively similar (P = 0.180; Fig. 2b). The average number of medications for all antidepressants and antipsychotics were also reported in Table S1. Additionally, the average dose for each medication was reported in Table S4; however, these data were not further analyzed due to the low n per medication in each cohort.

### Efficacy comparison between TAU and PGx revealed similar levels of improvement

**Fig. 3:**
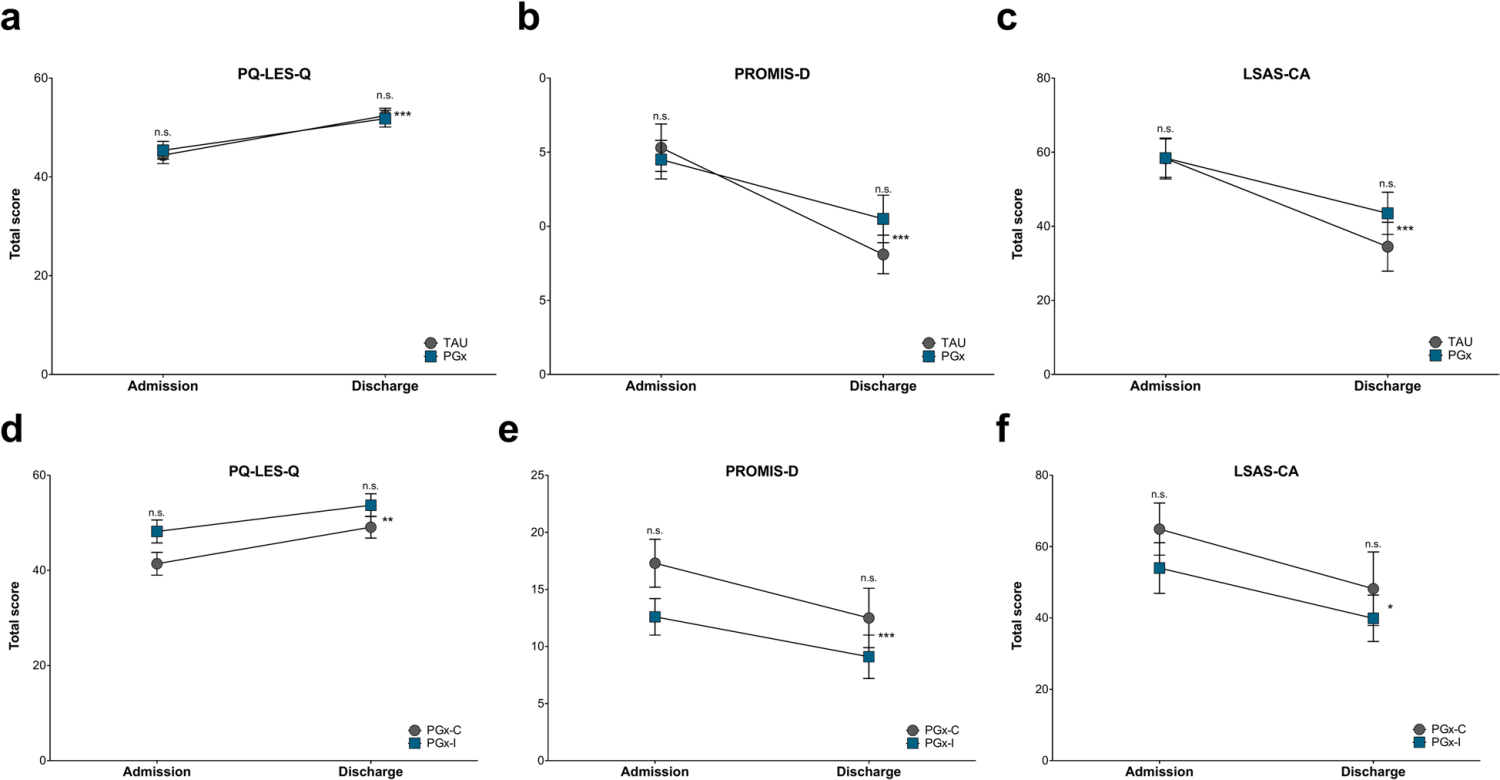
Quality of life, anxiety and depression improved between admission and discharge for both the TAU and PGx groups. **a** PQ-LES-Q scores at admission were similar between the TAU and PGx groups (P = 0.664). Quality of life for both groups improved to reach similar levels at the time of discharge (P = 0.805), with both groups reporting average life satisfaction. **b** LSAS-CA scores at admission were also similar between the TAU and PGx groups (P > 0.999). Social anxiety for both groups improved to similar levels at the time of discharge (P = 0.339), with both groups reporting mild levels of social anxiety. **c** PROMIS-D scores at admission did not differ between the TAU and PGx groups (P = 0.671). Depressive symptom severity for both groups improved to reach similar levels at the time of discharge (P = 0.223), with both groups reporting none to slight levels of depressive symptoms. **d** PQ-LES-Q scores at admission were similar between PGx-C and PGx-I (P = 0.059), with quality of life improving for both subgroups at discharge (P = 0.188) and reporting average life satisfaction. **e** LSAS-CA scores at admission were similar between PGx-C and PGx-I (P = 0.309), with both subgroups reporting improvements in social anxiety to mild levels by discharge (P = 0.483). **f** PROMIS-D scores at admission did not differ significantly between PGx-C and PGx-I (P = 0.079), with depressive symptom severity improving for both subgroups to none to slight levels by discharge (P = 0.295). *P < 0.05; **P < 0.01; ***P < 0.001; n.s. = not significant. Data reported as mean ± SEM

Quality of life, anxiety and depression assessments were utilized to evaluate whether PGx testing improved outcomes compared to TAU. Between admission and discharge the co-primary efficacy variable, the PQ-LES-Q score, showed that both groups had improved from low to average life satisfaction (P < 0.001; Fig. 3a). The PROMIS-D scores similarly demonstrated that both groups improved from none to slight levels of depressive symptoms between admission and discharge (P < 0.001; Fig. 3b). Social anxiety scores, as measured with LSAS-CA, also showed that both groups improved from moderate to mild levels of social anxiety between admission and discharge (P < 0.001; Fig. 3c). The within-group comparison showed improvement from admission to discharge for the TAU and PGx groups for skin-picking (P < 0.001), hair pulling (P = 0.017) and nail biting (P < 0.001) (Table 1). Scores for both the TAU and PGx groups across these measures did not differ at admission and discharge (Table 1).

Additional measures of the CASI (P < 0.001), PSWQ (P < 0.001) and CY-BOCS (P < 0.001) revealed symptom improvement for both the TAU and PGx groups between admission and discharge across each measure (Table S1). When scores for TAU and PGx were compared at discharge, the TAU and PGx groups reached similar levels of improvement of anxiety sensitivity and worry, as measured by the CASI and PSWQ assessments, respectively. However, the TAU group improved to subclinical level (7.2 ± 1.3) while the PGx group continued to report a mild level of OCD symptoms (11.9 ± 1.6) (P = 0.028; Table S1).

### PGx congruency comparison

To better understand how PGx results may be integrated into clinical decision-making for medications, we next investigated outcomes between the individuals prescribed congruent medications compared to those prescribed one or more incongruent medication(s). Individuals either remained on an incongruent psychotropic medication (n = 26) or they either remained on, or switched to, a congruent medication (n = 20) following the PGx test results for the remainder of their treatment.

Quality of life, anxiety and depression were compared between individuals taking congruent medications (PGx-C) and those taking incongruent (PGx-I) medications. Between admission and discharge the co-primary efficacy variable, the PQ-LES-Q score, showed that both subgroups had improved from low to average life satisfaction (P = 0.001; Fig. 3d). The PROMIS-D scores suggested that both subgroups improved from none to slight levels of depressive symptoms between admission and discharge (P < 0.001; Fig. 3e). The LSAS-CA also showed that both subgroups improved from moderate to mild levels of social anxiety between admission and discharge (P = 0.015; Fig. 3f). The RBBS scores showed improvement from admission to discharge for the PGx-C and PGx-I subgroups for skin-picking (P = 0.047) and hair pulling (P = 0.038), but not for nail biting (P = 0.068) (Table 2). Scores for both the PGx-C and PGx-I subgroups across all three measures did not differ at admission and discharge (Table 2).

Additional measures of the CASI (P = 0.086), PSWQ (P = 0.052) and CY-BOCS (P < 0.01) revealed symptom improvement between admission and discharge for only the CY-BOCS (Table S1). When compared at discharge, the PGx-C and PGx-I subgroups reached similar levels of improvement of anxiety sensitivity, worry and OCD symptoms as measured by the CASI and PSWQ and CY-BOCS assessments, respectively (Table S3).

### CYP2D6/CYP2C19-restricted medication management using CYP2D6 and CYP2C19

Exploratory analysis focused on CYP2D6 and CYP2C19, with individuals prescribed medications incongruent to their CYP2D6 and CYP2C19 metabolizer status (CYP2D6/CYP2C19–I) averaging 3.4 ± 0.3 psychotropic medications at discharge compared to 3.2 ± 0.2 for those prescribed congruent psychotropic medications (CYP2D6/CYP2C19-C) (P = 0.521; Table S2).

Additional exploratory analysis measuring quality of life, anxiety and depression assessments was next analyzed to evaluate how CYP2D6/CYP2C19-C and CYP2D6/CYP2C19-I subgroups changed over the course of treatment. The co-primary efficacy variable, the PQ-LES-Q score, indicated that both subgroups had improved from low to average life satisfaction; however, no differences were observed between the CYP2D6/CYP2C19-C and CYP2D6/CYP2C19-I subgroups at admission (P = 0.727) or discharge between subgroups (P = 0.292; Table S2). The PROMIS-D scores similarly demonstrated that both subgroups improved from none to slight levels of depressive symptoms between admission and discharge, but did not differ at admission (P = 0.442) or discharge between subgroups (P = 0.660; Table S2). LSAS-CA scores suggested that the CYP2D6/CYP2C19–C and CYP2D6/CYP2C19-I subgroups were similar at admission (P = 0.505) and discharge (P = 0.486; Table S2). Additional analyses for RBBS subgroups for skin-picking, hair pulling and nail biting indicated similar levels of improvement between subgroups (Table S2), as well as for PSWQ, CY-BOCS and the CASI (Table S3).

## Discussion

### Implications of research findings from the current study

This study explored the potential role of incorporating PGx testing into clinical workflows to optimize medication management for individuals with ASD. Notably, 57% of the study population admitted on at least one medication with a potential gene–drug interaction. With co-occurring conditions frequently affecting persons with ASD, medication regimens commonly include the use of SSRIs, antipsychotics, and psychostimulants [39]. Polypharmacy in the current study was common, with over half of individuals were prescribed three or more psychotropic medications at the time of admission, and approximately half of those individuals were prescribed at least one incongruent medication. Prescribers did appear to utilize the recommendations provided within the PGx report to either switch medications or adjust dosing those admitting on incongruent medications. However, these adjustments did not result in significant changes for any outcomes evaluated in the current study, and therefore the overall utility of PGx was uncertain.

Polypharmacy is common with ASD in general [36], and remains a challenge that patients and prescribers must navigate to minimize side effects and drug-drug interactions, and to promote medication adherence. As individuals with ASD age into adulthood, the polypharmacy concerns grow as non-psychiatric co-occurring conditions develop [15, 30]. While we hypothesized that individuals taking congruent medications would be prescribed fewer medications compared to those prescribed incongruent medications, the data did not support this. Overall, prescribers frequently changed medications or adjusted dose when the PGx report indicated an increased potential for gene–drug interactions, switching to alternatives with a decreased likelihood of gene–drug interactions. Given the high level of polypharmacy in this population, there is an opportunity to integrate PGx testing in the medical workup conducted at admission to reduce the number of incongruent medications.

This study also highlights that PGx testing is only one of multiple factors guiding medication management decisions. The retrospective study design makes it unclear to what degree decisions were informed by guidelines and algorithms, historical family data, patient and family preference, prescriber preference, insurance reimbursement, or careful use of the PGx report to avoid potential incongruent medications. One of the more important findings of the study was that all individuals improved similarly irrespective of access to PGx testing. To further explore the potential of PGx testing for individuals with ASD, we compared the impact of the combinatorial panel with PK-focused panels.

### Did evaluating CYP2D6 and CYP2C19 improve utility for clinical decision support over the combinatorial PGx panel?

PGx remains of great interest when prescribing psychotropic medications, yet there is no consensus as to which test or specific genes provide the most utility as part of the psychotropic medication selection decision. Some stakeholders advocate for a combinatorial test report [18], inclusive of a range of PK- and PD-related genes, while others focus on specific genes, such as CYP2D6, CYP2C19, CYP2B6 and others [7, 24, 34]. Previous comparisons between both approaches reported the combinatorial PGx test outperformed individual genes with major depression [38], however, little is known about this comparison in ASD. To better understand how these testing approaches can be used for children and adolescents with ASD, the current study evaluated both a comprehensive PGx panel and a PK-focused panel inclusive of only CYP2D6 and CYP2C19 to determine the impact on medication changes and assessment outcomes. The resulting data did not support one approach over another. Taken together with the complex factors informing medication changes, it may be most impactful for prescribers to order the combinatorial PGx panels, as the recommendations when narrowing to CYP2D6 and CYP2C19 were similar to the more comprehensive commercial product. Commercial PGx products also tend to have short turnaround times, which can be helpful in high acuity settings. These panels typically report the metabolizer status of specific genes, such as CYP2D6 and CYP2C19, allowing prescriber discretion on which clinical decision tool to utilize as part of the overall medication selection decision.

### Clinical implications

The study’s co-primary outcome of polypharmacy rate did not differ between treatment groups. Indeed, medication selection and dosing decisions are complex and multifactorial. Prescribers must navigate guidelines and algorithms, family history, past medication trials, co-occurring conditions, allergies, patient and family preferences, insurance reimbursement and other factors. Disentangling the impact of the results from PGx reports in such a complex clinical decision-making context is challenging, particularly when patient outcomes were analyzed retrospectively. Moreover, the retrospective nature of the study did not allow us to understand how the PGx tests were used by prescribers and how the data were weighted in decision-making, e.g., a moderate versus significant potential gene–drug interaction and the cost–benefit of PGx testing.

Clinical assessment outcomes were modest across all treatment groups. The PQ-LES-Q showed improvement from low to average quality of life across all groups. Improvements were also observed for depressive symptoms, social anxiety and repetitive behaviors, regardless of treatment group. The modest overall improvements for all treatment groups, lack of separation between any treatment groups when stratified by combinatorial PGx test and medication congruency (PGx-C versus PGx-I; CYP2D6/CYP2C19-C versus CYP2D6/CYP2C19-I), and complexity of medication decision-making suggest that longitudinal prospective trials that incorporate long-term follow-up are necessary. To more comprehensively assess the role of pharmacogenomic testing in this population, future longitudinal studies should incorporate detailed prescribing histories, data on non-psychotropic medication use, more granular classification of CYP2D6 and CYP2C19 metabolizer status to inform medication selection, surveys of prescribers to evaluate how PGx results influenced clinical decisions, documentation of side effects, and the inclusion of ASD-specific outcome measures.

### Limitations

Due to the retrospective nature of the study, medication compliance was reliant on patient self-report and prescription refills, and the history of failed medication trials were not evaluated. The sample size may have limited the detection of statistical differences, particularly within subgroups. While the assessments used for the current study are validated in pediatric populations, they were also used for individuals up to the age of 20 within the treatment program. Additionally, the relatively brief length of stay, which averaged under 40 days, decreased the likelihood of observing medication-related differences in assessment outcomes. Communication and sensorial disorders, which are prevalent in this population, were not evaluated in the current study and as part of the treatment program. The classification of medications based on CYP2D6 and CYP2C19 metabolizer status may not fully align with specific recommendations that consider additional genes (e.g., CYP3A4) or specific metabolizer statuses (e.g., ultrarapid metabolizer), potentially influencing the findings. Although the study allowed for the inclusion of any pharmacogenomic (PGx) test, only the GeneSight PGx test was used by prescribers, limiting the generalizability of findings to other PGx tests. Only psychotropic medications were evaluated in the current study, leaving the possibility that drug–drug interactions may have occurred with additional prescribed medications that could affect prescribing decisions related to these medications. Although adverse effects were monitored as part of standard of care for all individuals in the program, they were not systematically evaluated or compared between groups in this study.

## Conclusion

The current study evaluated the utility of PGx testing for individuals with ASD in a high-acuity depression and anxiety program. Medication selection or dose changes appeared to be adjusted based on the PGx results, although it is unclear if prescribers used it as a clinical decision-making tool. No benefit in clinical outcomes was observed when assessing congruency, polypharmacy, quality of life, symptom severity or other variables. However, the level of evidence presented in this study does not invalidate the potential benefit of PGx for individual cases or the need for a prospective, definitive clinical trial to more clearly establish its utility, as is recommended by AACAP.

## Supplementary Information

Supplementary Material 1.

Supplementary Material 2.

Supplementary Material 3.

Supplementary Material 4.

## Data Availability

The data used in this retrospective study were obtained from the electronic health record system for Rogers Behavioral Health and are not publicly available due to patient privacy. Researchers interested in accessing the data for further analysis may contact the corresponding author to discuss potential data sharing under the approval of the Institutional Review Board and with appropriate data access agreements in place.

## Abbreviations

AACAP: American Academy of Child and Adolescent Psychiatry
APA-COR: American Psychiatric Association Council of Research
ADHD: Attention-Deficit/hyperactivity disorder
ASD: Autism Spectrum disorder
CASI: Childhood Anxiety Sensitivity Index
CY-BOCS: Children's Yale-Brown Obsessive–Compulsive Scale
CPIC: Clinical Pharmacogenetics Implementation Consortium
EHR: Electronic health record
FDA: Food and Drug Administration
IRB: Institutional Review Board
IOP: Intensive Outpatient Program
LOS: Length of stay
LSAS-CA: Liebowitz Social Anxiety Scale for Children and Adolescents
OCD: Obsessive-Compulsive Disorder
PHP: Partial Hospitalization Program
PROMIS-D: Patient-Reported Outcomes Measurement Information System Pediatric Depressive scale
PQ-LES-Q: Pediatric Quality of Life Enjoyment and Satisfaction Questionnaire
PSWQ-C: Penn State Worry Questionnaire for Children
PD: Pharmacodynamic
PGx: Pharmacogenomics
PK: Pharmacokinetic
RBBS: Repetitive Body-Focused Behavior Scale
SSRI: Selective Serotonin Reuptake Inhibitor
SNRI: Serotonin-norepinephrine Reuptake Inhibitor
SEM: Standard Error of the Mean
TAU: Treatment as Usual
